# The Treatment with Interleukin 17 Inhibitors and Immune-Mediated Inflammatory Diseases

**DOI:** 10.3390/cimb44050127

**Published:** 2022-04-26

**Authors:** Laura Țiburcă, Marius Bembea, Dana Carmen Zaha, Alexandru Daniel Jurca, Cosmin Mihai Vesa, Ioana Adela Rațiu, Claudia Maria Jurca

**Affiliations:** 1 Faculty of Medicine and Pharmacy, University of Oradea, 1 December 10 Square, 410087 Oradea, Romania; laura_tiburca@yahoo.com (L.Ț.); v_cosmin_15@yahoo.com (C.M.V.); ratiu_ioana@yahoo.com (I.A.R.); claudiajurca70@yahoo.com (C.M.J.); 2 “Dr. Gavril Curteanu” Clinical Hospital Regional Center of Medical Genetics Bihor, 410469 Oradea, Romania; bembea13@yahoo.com

**Keywords:** IL-17 inhibitor, inflammatory intestinal disease, Crohn’s disease, ulcerative colitis

## Abstract

IL-17 inhibitors (IL-17i) are medicines used to treat dermatological and rheumatic diseases They belong to a class of medicines called biological disease-modifying anti-rheumatic drugs (bDMARDs). This class of drugs has had a major impact on the therapy of autoimmune diseases, being much safer and more effective than treatment with small molecules. At the same time, they have highly beneficial effects on skin and joint changes, and their efficacy has been extensively monitored and demonstrated in numerous clinical trials. More and more such drugs are still being discovered today to ensure the best possible treatment of these patients, but more frequently and relatively constantly three agents are used. Two of them (Secukinumab and Ixekizumab) inhibit IL-17A directly, and the third, Brodamulab, inhibits the IL-17A receptor. Although they are extremely effective in the treatment of these diseases, sometimes their administration has been associated with paradoxical effects, i.e., there is an exacerbation of the inflammatory process. Tough, clinical trials of IL-17i have described cases of exacerbation or even onset of inflammatory bowel disease (IBD), such as Crohn’s disease and ulcerative colitis, after administration of these drugs in patients previously diagnosed with psoriasis (PS), psoriatic arthritis (PsA), or ankylosing spondylitis (AS). The pathophysiological mechanism of action is not well understood at present. One explanation would be that this hyperreactive inflammatory process would be triggered by Interferon 1 derived from dendritic plasma cells. Even though there are many reports in the recent literature about the role of IL17i in the onset of IBD, conclusions of studies do not converge. Some of them show an increased incidence of IBD in patients treated with IL17i, while some others affirm their safety of them. In the near future we will surely have more data emerging from ongoing meta-analyses regarding safety of use IL17i in patients who are at risk of developing IBD. Clinical and paraclinical evaluation (inflammatory intestinal markers) are carefully advised before recommending treatment with IL-17i and after initiation of treatment, and prospective surveillance by clinical and biomarkers of patients treated with IL-17i is absolutely essential to capture the onset of IBD.

## 1. Introduction

Interleukin-17 (IL-17) is one of the well-studied cytokines as an important player in the mammalian immune system. IL-17 was originally thought to be produced by CD4 Th17 but is now known to be produced by a variety of other cells including macrophages, dendritic cells, CD8 Tc17, lymphoid tissue inducer, and γδ-T [1]. Although this cytokine plays an essential role in many infectious diseases, it promotes inflammatory pathology in autoimmunity and other settings. Higher levels of IL-17 were found in target tissues in the lungs in subjects with moderate-to-severe asthma, synovial from rheumatoid arthritis patients, inflamed colon, skin, and kidneys, demonstrating its role in local tissue damage.

Many studies have focused on how IL-17-producing cells are formed. However, the mechanisms by which IL-17 achieves its effects, for the benefit or harm of the host, are due in large part to the induction of a new gene expression. Although many genes targeted at IL-17 are common in various disease states, in some cases the effects of IL-17 vary depending on the target cell, infectious site, or pathogen. The gene products induced by IL-17 include cytokines (IL-6, granulocyte-colony-stimulating factor, tumor necrosis factor-α), chemokines (CXCL1, CXCL2, CCL20, among many others), and inflammatory factors such as acute phase proteins, complement and antimicrobial proteins (defensins, mucins). Different cell types react differently to IL-17 in terms of target gene expression, with significant differences observed in mesenchymal and epithelial cells compared with hematopoietic origin cells [2].

Immune-mediated inflammatory disease is a group of conditions that present disturbances in common inflammatory pathways. It includes ankylosing spondylitis (AS), psoriasis, psoriatic arthritis (PA), rheumatoid arthritis (RA), type 1 diabetes, and other inflammatory conditions. Although the etiology of these conditions is insufficiently elucidated, advances in research have demonstrated an imbalance in inflammatory cytokines.

Monoclonal antibody therapies are modern and widely used for the treatment of many chronic inflammatory diseases. Interleukin 17 inhibitors (Ixekizumab, Secukinumab, Brodalumab) are used successfully in the biological treatment of psoriasis, PA, AS, RA, but at the same time mediate the immune response against bacteria and fungi [3]. Since IL17i have been approved, the possibility of a paradoxical effect occuring in some cases was noticed, which means the exacerbation or new onset of a disease that was supposed to be cured by this medication. Assessing the risk of paradoxical effects in the treatment of any of these conditions is difficult due to the possible interrelationship between the diseases of this group. Thus, from the beginning, there is a 2.9× higher risk that patients diagnosed with psoriasis will develop inflammatory intestinal disease (IBD) or have a subclinical form of the disease compared to the general population; a patient with Crohn’s disease has a 7× higher risk of developing psoriasis [4]. The pathophysiological mechanisms underlying the exacerbation or de novo occurrence of IBD are not yet sufficiently known, but this hyperreactive inflammatory process could be triggered by Interferon 1 derived from dendritic plasma cells [5]. Because of this, there are no clear treatment guidelines for these patients; screening to prevent or exacerbate inflammatory intestinal disease before starting treatment with IL-17 inhibitors is extremely important. It is currently unclear whether this category of drugs causes de novo IBD or whether the onset of IBD in these individuals is due to their genetic predisposition as they already had a subclinical form of IBD [6,7]. Other adverse events are infections of upper respiratory tract, including candidiasis, oral herpes, headache, neutropenia, and diarrhea.

The aim of the article is to highlight the role of IL-17 inhibitors in the occurrence of IBD in patients with psoriatic arthritis, AS, and others inflammatory conditions based on the latest data published in the literature. There are also some practical recommendations useful for clinicians at the point of choosing treatment with IL17i in order to minimize the risk of getting an exacerbation or new-onset of IBD.

## 2. Materials and Methods

The item selection process is illustrated in the PRISMA diagram. We considered full-text articles and relevant abstracts based on relevance and novelty.

We performed a search on the relevant literature and selected scientific publications addressing the correlation between the IL-17 inhibitors treatment and inflammatory bowel disease. The PubMed, Web of Science, and Scopus databases were searched for relevant information related to this topic. The method used to search for relevant information was done with the help of the “AND” operator and specific keywords: “IL-17 inhibitors” AND “inflammatory intestinal disease” (Table 1). The articles considered eligible were initially selected based on both their title and abstract; then, a complex analysis of their content was performed, and the most relevant and informative data and results were extracted.

## 3. Interleukin Family

Since the year 2000, the members of this family and the way they and their recipients work together have been understood to provide answers to how immunity to infections is directed. After 2005, when a third subset of Th lymphocytes, Th17 cells, was identified, in addition to the already known Th1 and Th2 cells, the scientific interest in this cytokine suddenly increased.

IL-17 belongs to a family of cytokines comprising six entities (IL-17A-IL-17F) and five receptors (IL-17RA-E). IL-17A is the most intensively studied. Il-17F has a quasi-similar structure to IL-17A (55%) and is frequently co-expressed with IL-17A, but its action on proinflammatory genes (CXCL1, IL6, CCL2, CCL7, and Matrix Metalloproteinases (MMP)) is less expressed [8,9]. The other components of the family are similar to IL-17A (23–29%), IL-17E being the most different from IL-17A (16% concordance). IL-17D, E and F are expressed in epithelial cells, but their precise proinflammatory role is not fully elucidated [10,11,12]. These cytokines are essentially disulfide-linked homodimers, and sometimes IL-17A and F can form heterodimers [13]. IL-17F was demonstrated to be expressed in the airway of patients with asthma and its expression level was correlated with disease severity.

IL-17 is one of the most intensely and best studied cytokines due to its important role in inflammatory diseases. Their secretion is mainly due to Th17 cells (signature cell) but in addition to these, other cells of the immune and adaptive system such as IL-17 secretion are involved: CD8 + T cells, γδ-T cells, natural killer T cells, dendritic cells, macrophages lymphoid cells [14,15]. In turn, IL-17 influences the action of other immune system cells (B cells, antigen presenting cells) to induce the expression of chemokines, chemokine receptors, cytokines, and metalloproteases.

**Figure 1:**
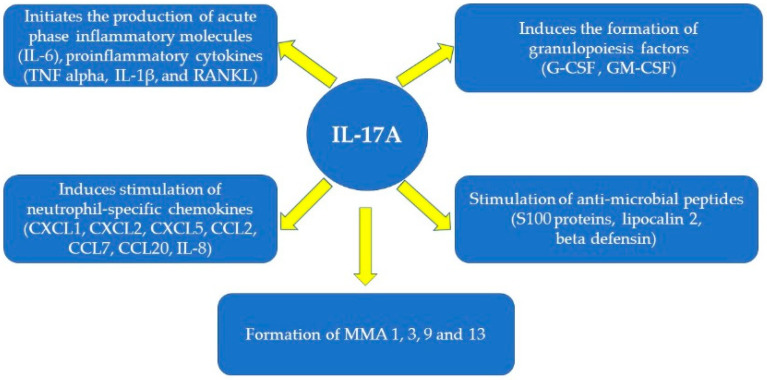
Roles of IL-17A.

It is now well established that Th17 cells responsible for producing IL-17 are involved in maintaining good intestinal function by regulating the immune response against autoantigens or intestinal pathogens due to T-cell binding (Treg). In the past, the role in IBD pathogenesis was exclusively attributed to Th1 cells and Th2 cells [16]. Due to the decrease in cytolysis function, Th17 cells produce large amounts of IL-17A, IL-17F, IL-26, IL-8, IL-10, interferon γ TNF-α, and IL22. The latter are important in the homeostasis of the intestine by maintaining its full barrier function, repairing the lesions while providing protection against the fungi of the intestinal microbiome [17,18,19]. Actions of Il-17A can be seen in Figure 1 [2,20,21].

Overall, these pro-inflammatory cytokines contribute to the development of RA and reestablish a chronic inflammatory state with a loop of positively stimulating responses when IL-17-induced IL-6 maintains Th17 T-cell levels [22]. It also activates cyclooxgenase-2 pathway, resulting in prostaglandin E2, which promotes inflammation in a number of ways including vasodilatation [23].

**Figure 2:**
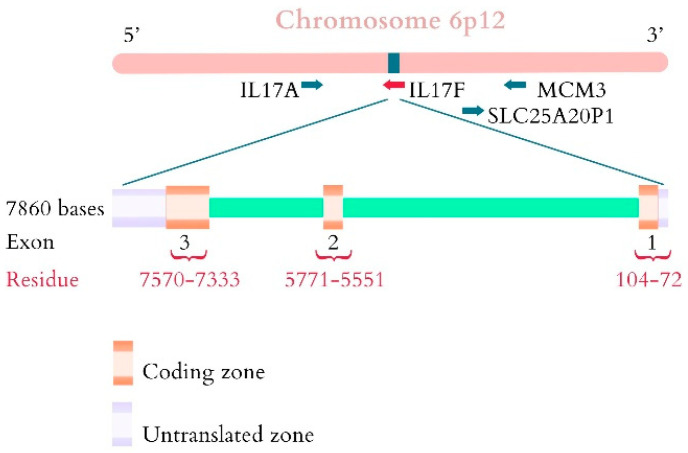
The IL-17A gene, located on the short arm of chromosome 6, contains 7860 bases, 3 exons, and 2 exons.

The gene encoding IL-17 was cloned in 1993 from a cytotoxic mouse T lymphocyte hybridoma cDNA library and is located at the short arm of chromosome 6 (6p12.2) [24]. The gene contains 7.86 kb and consists of 3 exons, the coding region, and 2 introns. The encoded protein contains 163 amino acids (Figure 2). The latter is activated by T cells, but can also be secreted by macrophages, dendritic cells, natural killer (NK), and lymphoid tissue inducing cells.

## 4. The IL-17 Receptor Family

**Figure 3:**
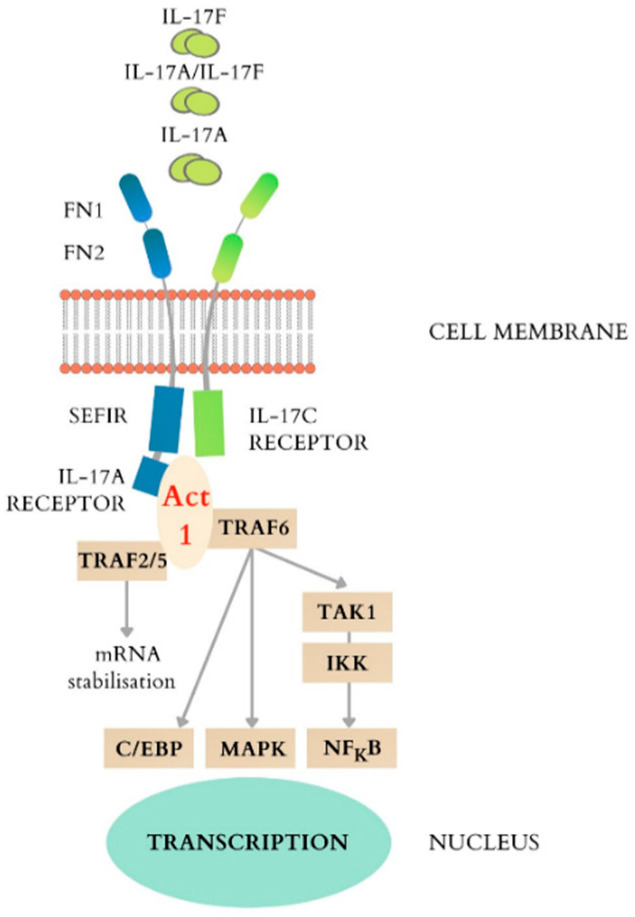
The IL-17 cytokines and their receptors, the main signaling events downstream of the activation of IL-17A receptor.

The first IL-17 receptor was identified in 1995 [25,26]. Until the year 2000, its role was uncertain. Receptors are transmembrane proteins with unique properties. Its extracellular component consists of two domains similar to fibronectin III with a role in the interaction between protein and ligand. The cytoplasmic part of the receptor contains, at the C-terminus, the sequence SEFIR (similar expression of fibroblast growth factor genes and IL-17Rs), which is related to the TIR domain (toll/interleukin-1 receptor) found in IL-1 and the family of toll-like receptors [20,25]. SEFIR binds to the Act1 adapter protein which will cascade several signaling pathways. Initially recruiting recombinant human TNF receptor-associated factor 6 (TRAF6) that mediates signals received from receptors, it further activates in response to the proinflammatory cytokines IkappaB kinase (I κ K), ultimately leading to nuclear transcription of NFκB and activation of the genes targeted by it [27,28,29]. TRAF6 is also involved in the activation of the mitogen-activated protein kinase (MAPK) pathway. TRAF2/5, on the other hand, is involved in the maintenance of stability of the RNA molecule by sequestering the disintegration factor and by activating the RNA binding proteins [30,31]. In essence, this signaling pathway linked to the IL-17A receptor has two major effects: the stability of the RNA molecule and the promotion of the process of nuclear transcription of inflammatory genes (Figure 3).

## 5. The Role of IL-17 in Joint Inflammation and Bone Homeostasis

Persistence of inflammation can lead to impaired bone homeostasis. Normal bone function is under the control of three tumor necrosis factor (TNF) receptor proteins: nuclear factor-κB ligand activator (RANKL), nuclear factor κB receptor activator (RANK), and osteoprotegerin (OPG) [32]. All these factors play an important role in differentiating and forming bone. Osteoclasts have the role of adhering to the extracellular matrix; subsequently, they secrete lytic enzymes that degrade the matrix in an extracellular compartment. The mechanisms of osteoclastogenesis and bone resorption activation, as well as how hormonal stimulation influences bone structure, have been fully understood with the discovery of the RANK signaling pathway. Osteoclast formation is initiated by ligand binding (RANKL) to its receptor (RANK) in the osteoclast precursor; at the same time, OPG blocks osteoclastogenesis by inhibiting the interaction of the receptor activator thus having a protective role.

Boyle et al. in 2003, identified a number of 24 genes involved in osteoclast inactivation and in the positive or negative regulatory mechanism of osteoclastogenesis [33]. Mutations in these genes prevent the formation of osteoclasts and the consequences are increased bone and cartilaginous mineralization (osteopetrosis).

IL-17 has osteoclastogenic action by stimulating the expression of RANKL activator by osteoblasts and other stromal cells [34]. At the same time, IL-17A induces the expression of matrix metalloproteinases in fibroblasts, and endothelial and epithelial cells promote the destruction of both connective tissue and bone [33,35]. Decreased levels of IL-17A and IL-22 improve joint function. In patients with AS, serum Th17 levels are elevated [6,36,37], but treatment with their inhibitors has moderate therapeutic responses [38,39,40]. At the same time, Th17 was identified in joint tissue samples collected from patients with AS so that treatment with IL-17i was recognized as a therapeutic alternative [41,42].

## 6. The Role of IL-17 in Intestinal Inflammation

Il-17A, together with IL-23 through their proinflammatory action, plays an important role in mediating intestinal inflammation in IBA and also in other autoimmune diseases. In Romania, Lucaciu et al. in 2021 showed elevated values of Il-17A and IL-23 in the serum of 62 patients diagnosed with IBD [43].

Increased IL-17 levels in the lamina propria and in intestinal inflammation have been reported in various studies, including colorectal cancer tissues together with cellular energy metabolism changes [44]. The first study to show a correlation between elevated levels of IL-17A and IBD was that of Fujino et al. in 2003, followed by other reports [45,46,47,48]. The link between IL-23 and IBD has been demonstrated by the increased production of IL-23 by macrophages present in the lamina propria in Crohn’s disease and not ulcerative colitis by Fuss et al. [49]. Increased values are also found in patients with arthritis and sacroiliitis. Other studies have shown the opposite: elevated IL-23 levels are strictly related to the severity and duration of the disease, but only in ulcerative colitis [45]. IL-23R receptor polymorphisms play an important role in the development of intestinal inflammation [50,51]. Einarsdottir et al. have shown that the Arg381Gln allele provides three times greater protection against ulcerative colitis [52].

The subset of ILC3 cells also produce IL-17A in patients with AS and IBD. These cells are found in the intestinal mucosa in healthy people with a protective role. Their number has been shown to be increased in patients with AS who also associate with ileal inflammation. The hypothesis that ILC3 plays a role in the etiology of IBD has thus been raised, but this is not clearly proven today [53,54].

As elevated levels of IL-23, Th17 cells and IL-17 are found in the serum, intestinal mucosa, plasma of patients with CD and UC, and the same Th17 cells and IL-17 have been shown in animal specimens of IBD [55]. More than that, in a dextran sulfate sodium colitis mouse model, blocking of IL-17 activity results enhanced the expression of tumor necrosis factor (TNF)-α, interferon (IFN)-γ and IL-6, and the infiltration of T cells (CD4+ Th cells) and granulocytes–monocytes in the gut mucosa, finally resulting in the progression of colitis. In the same model, it has demonstrated recombinant IL-17, which attenuated the effect of anti-IL-17 antibody [56,57].

Chronic inflammatory intestinal disease is extremely common: the prevalence in 2017 was 6–8 million cases globally [58]. It is a disease with multifactorial polygenic transmission in its determinism, intervening in addition to susceptibility genes and environmental factors, the deficient immune system, but also the alertness of the intestinal microbiome [16,59]. It has been shown that some patients with a genetic predisposition are at an increased risk of developing de novo IBD when inhibiting IL-17.

It includes two major entities: Chron’s disease and ulcerative colitis. The first affects the entire gastrointestinal tract, from the mouth to the anus, and causes abdominal pain, weight loss, and changes in intestinal transit. The latter entity especially affects the colon and rectum, causing the appearance of the bloody stool [18,60]. The approach to diagnosis is multidisciplinary, as is the effectiveness of treatment [61].

When initiating treatment with IL-17i, a thorough medical history is extremely important. We must insist on a clinical examination being as correct and complete as possible on the family history, but also on previous digestive (gastrointestinal) manifestations. The patient should also be monitored throughout the treatment period, but also afterwards (one week to 4 years post-therapy) [4]. Gastrointestinal symptoms and chronic inflammation of the intestine after treatment with IL-17 inhibitors used in the treatment of psoriasis, PA, AS have been reported in the literature. However, there is currently no study that accurately and clearly highlights the correlation between the onset of IBD and this therapeutic class [62,63].

**Figure 4:**
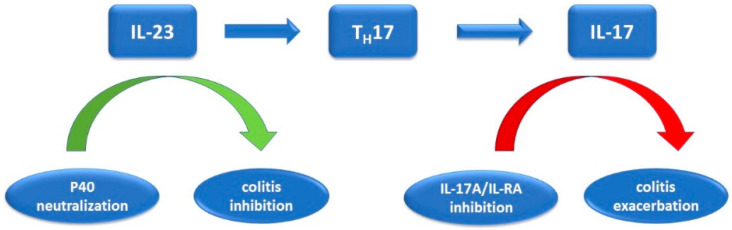
Effects of IL-23 and IL-17 inhibitors on colitis: IL-23 leads to Th17 synthesis, which in turn stimulates the release of IL-17; inhibition of IL 23 and IL-17 leads to two paradoxically different reactions.

Hohenberger et al. studied the association of inhibition of IL-17 with exacerbation of colitis correlating the evidence obtained in rodents with those in clinical trials. They noted that inhibition of IL-17 in the presence of intestinal inflammation worsens the disease even more [64,65]. It has been hypothesized that there are some patients in whom the genetic predisposition associated with the gut microbiome may be at increased risk of developing IBD during treatment with IL-17i. If Il-17 inhibition increases the inflammation gut with an increased risk in its occurrence, IL-23, through maintaining and expansion of the Th17 cells line, has positive feedback. The IL-23 blockade has positive effects in IBD without altering the intestinal barrier (Figure 4).

## 7. IL-17 Inhibitors and Their Role in Inflammatory Bowel Disease

**Figure 5:**
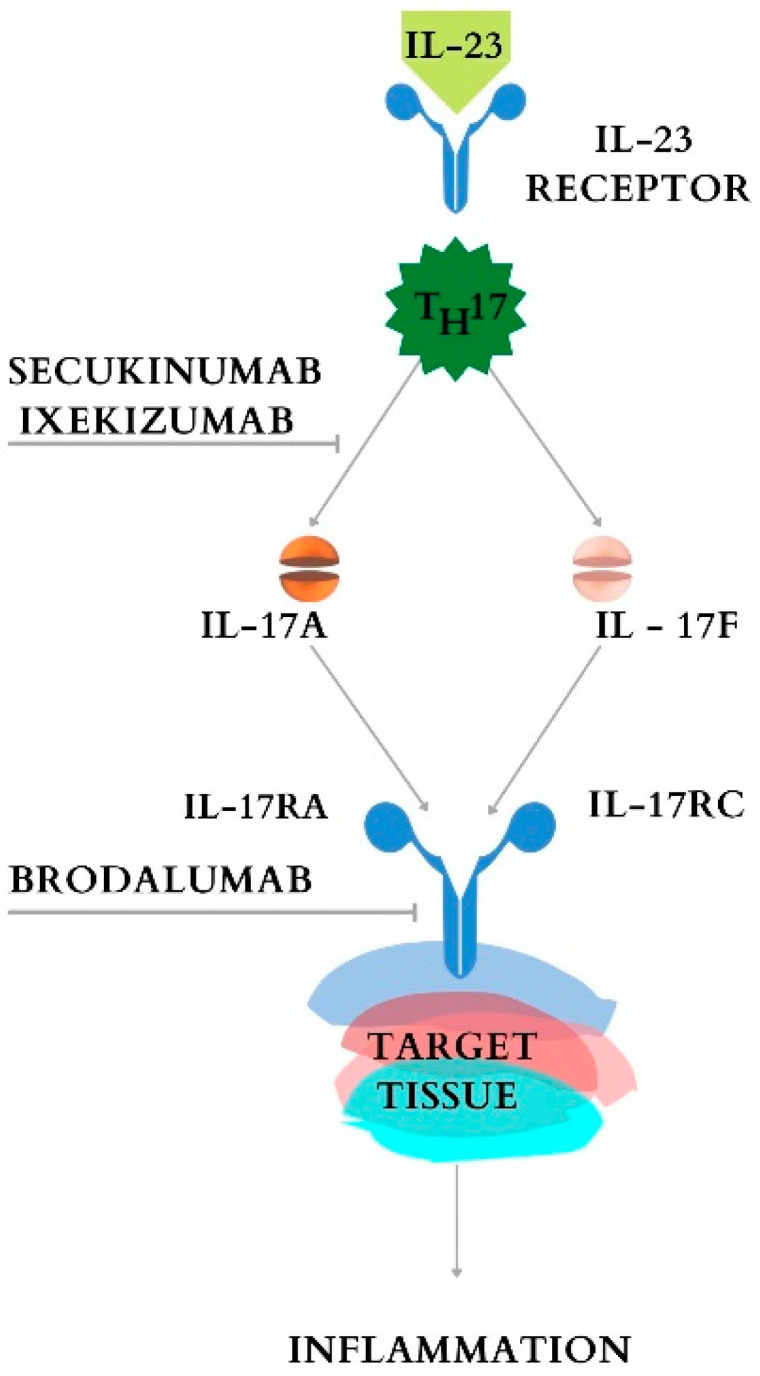
Action of IL-17 inhibitors: IXE and SEC blocks the IL-17A; Brodalumab blocks the receptor of IL-17A (IL-17RA).

The best-known anti-IL-17 monoclonal antibodies are Secukinumab (SEC), Ixekizumab (IXE), and brodalumab (BROD), which are widely used in the treatment of IBD for psoriasis and ankylosing spondylitis. Although they have extremely favorable effects on dermatological and rheumatological conditions, their use in IBD has not been approved due to paradoxical effects after administration (accentuation of symptoms after a so-called curative treatment). The three IL-17 inhibitors have been shown to be effective in Phase 3 of the clinical trials in the treatment of moderate forms of psoriatic plaque, psoriatic arthritis, and AS [66,67], but have been associated with worsening of IBD, especially Crohn’s disease with placebo [68]. Their mode of action is shown in Figure 5.

### 7.1. Secukinumab

It is a monoclonal antibody that blocks the binding of interleukin 17A to the corresponding receptor. This mechanism inhibits the proinflammatory effects of IL-17A downstream. It was approved in 2015 by the US Food and Drug Administration for the treatment of psoriasis, and for the treatment of psoriatic arthritis and AS in 2016. The dose is two subcutaneous injections of 150 mg (300 mg), weekly for the first 4 weeks, and then every 4 weeks [63]. It is the most commonly used monoclonal antibody, and its effects on intestinal inflammation have been reported in many clinical trials. In a study of 59 patients with moderate Crohn’s disease, Hueber et al. found in 2012 that it not only blocks IL-17 ineffectively but is also associated with multiple side effects and even worsening of the disease [66].

Gomez et al. from a group of 127 patients with rheumatological and dermatological conditions followed for a period of 4 years (January–December 2017), showed that three patients developed IBD, namely Crohn’s disease (two patients diagnosed with AS, one with PA), after treatment with IL-17i [67].

In a retrospective study performed in 2020, Wright et al. set out to determine the incidence of IBD within a cohort of patients diagnosed with psoriasis, using the data in the patient’s electronic files. They analyzed patients were those who underwent treatment with IL-17i, those who received treatment only with Secukinumab, and those who did not receive treatment with IL-17i. Evaluation took place at 6 and 12 months, respectively. The incidence of IBD at 6 months was the following: of the 1821 patients treated with IL-17i, three developed IBD (0.16%), and of the 1246 patients treated only with SEC, three developed IBD (0.24%); IBD occurred in 239 patients of the total of 213,060 who have not received treatment with IL-17i (0.11%). At the first year, the incidence of IBD was 0.27% for the patients who received IL-17i, 0.32% for those being treated with SEC only, and 0.19% for those who did not receive treatment with IL-17i. The study’s conclusions were that there exist no statistical differences, neither at 6 nor at 12 months, between the patients treated with SEC and those who did not receive this treatment [68].

A meta-analysis of 38 randomized controlled trials (RCTs) looked at the occurrence of de novo IBD in 16,690 patients diagnosed with psoriasis, PA, and AS treated with IL-17i. The follow-up period was 60 weeks. Twelve cases of de novo IBD (Crohn’s disease five cases, ulcerative colitis seven cases) were identified in patients receiving treatment with Ixekizumab (four cases) and Secukinumab (eight cases); no adverse reactions were reported after treatment with Brodalmab [69,70]. Schreiber et al. through a joint analysis of 21 clinical trials enrolling 7355 patients treated with Secukinumab for psoriasis, PA, and AS, tracked the incidence of IBD in these patients. Patients treated with SEC were followed for five years for psoriasis and PA and for up to four years for AS. Of the 5181 patients with psoriasis, 68 were also diagnosed with IBD; of the 1380 with psoriatic arthritis, eight cases of IBD were diagnosed, and of the 794 patients with AS, 13 had IBD. It should be noted that in this study, a third of the patients had previously been treated with TNF alpha inhibitors [71].

Fauny et al. collected clinical cases of de novo inflammatory intestinal disease reported in the literature up to 2020 after treatment with Secukinumab and Ixekizumab. They identified 21 reported cases (19 after treatment with Secukinumab and 2 after treatment with Ixekizumab). Of this number, ten had ulcerative colitis, six had Crohn’s disease, and five had unidentified IBD. The diagnosis of IBD was confirmed in all patients by abdominal CT, Magnetic Resonance Enterography (MRE) and anatomopathological changes. Digestive symptoms had a variable onset: 1 week to 56 months from the start of treatment to 12.8 months. The recommended treatment for these symptoms is based on the administration of TNF inhibitors and corticosteroids, with Secukinumab being discontinued in all cases. The subsequent evolution was with the relief of symptoms [6]. Additionally, in 2020, Fieldhouse et al. collected literature articles published over a period of two years (2018–2019), identifying 20 publications on the onset of IBD after treatment with IL-17Ai, publications that described 27 patients diagnosed with psoriasis, PA, and AS [4]. Meta analysis studies and multicase report studies have shown efficiency and safety of IL17i, most of them focusing on reduced incidence of IBD in treated patients (Table 2).

In 2018, the European Medicines Agency (EMA) changed the results of the product characteristics (PRC) for Secukinumab by adding a warning about inflammatory intestinal disease. In 2019, with growing evidence in this regard, the EMA added “inflammatory intestinal disease” as drug side effects (ADRs), with the frequency estimated as “Rare” (uncommon).

### 7.2. Ixekizumab

Approved for administration by the US Food and Drug Administration in 2016, IXE is a monoclonal antibody that has the same mechanism of action as Secukinumab, blocking the binding of IL-17A to its receptor. The recommended therapeutic doses for the treatment of psoriasis are 160 mg (2 × 80 mg) injections subcutaneously in week 0, followed by the administration of 80 mg in weeks 2, 4, 6, 8, 10, 12, then 80 mg at 4 weeks. In PA and AS, the recommended doses are: 160 mg (two injections of 80 mg) subcutaneous injections at week 0 followed by 80 mg every 4 weeks.

In 2017, Griffiths et al. in a post hoc analysis, looked at the side effects of 4209 patients diagnosed with a moderate form of psoriasis with suspected IBD in 7 clinical trials. Of these, 19 reported IBD (15 new cases), but noted that an increased number of patients was not reported (IBD history was obtained on a voluntary basis). Two patients had symptoms of IBD, namely Crohn’s disease during the induction period [7]. Smith et al. reported a similar case, a patient with a moderate form of psoriasis, treated with IXE who showed symptoms of IBD on day 2 after the end of induction treatment [73,74].

In a phase 3 study by van der Heijde et al. the efficacy of IXE in the treatment of AS was tracked. More precisely, IBD Crohn’s disease was reported in only one patient out of a total of 164 who were followed for a period of 1 year. The joint analysis of 7 clinical trials looked at the short- and long-term efficacy of Ixekizumab in psoriasis; the incidence of IBD in these trials was low, with only one patient with newly diagnosed BC, an exacerbation of ulcerative colitis in a patient during the induction period, and 4 flares of maintenance colitis [72,75,76].

### 7.3. Other IL-17i

Brodalumab is a monoclonal antibody that acts through blocking the IL-17 receptor (IL-17R) [77]. It was recently introduced to the market and is strictly recommended for the treatment of psoriasis. As a newcomer, no cases of IBD are currently reported after its administration. In a Phase 2 study, Tartan et al. concluded that treatment with Brodalumab had not been shown to worsen digestive symptoms in a disproportionate number of cases and no significant efficacy was found, but no other side effects were reported [78].

Bimekizumab another monoclonal antibody in this category is only in the clinical trial phase and Netakimab is administered only in Russia [4,7]. Adverse effects were reported by 4.4% (9/206) of whole study population up to week 48 (1/206 [0.5%] receiving placebo and 3.9% (8/206) receiving bimekizumab). The most common adverse effects were nasopharyngitis (12.1%) and oral candidiasis in 4.9% of all bimekizumab-treated cases at week 48. No deaths, cases of new-onset inflammatory bowel diseases, or major adverse cardiac events were reported [79].

In the literature, three major epidemiological and pharmacovigilance studies are published. The first retrospective study noted is that of Orrell et al. in 2018, collecting data from various databases: Research on Adverse Drug Events and Reports and Northwestern Medicine Enterprise Data Warehouse. A representative group of over 5 million patients with Crohn’s disease and ulcerative colitis was included, following the patients who developed IBD after treatment with SEC and IXE. Patients who received SEC during January 2015–August 2017 and those who received treatment with IXE 2016 (January)–2017 (August) were reviewed. Conclusions of the study were that adverse events reported after administration of Secukinumab to the Food and Drug Administration Adverse Event Reporting System were insignificant. Similar therapeutic effects have also been questioned following the administration of both drugs and the occurrence of IBD (Crohn’s disease or ulcerative colitis) [80].

The second retrospective study was by Mohy-ud-din et al. in 2019, which looked at whether SEC increases the risk of developing IBD. The authors used the Explorys platform, which contains data from about 62 million electronic health records. The study concluded that the risk of developing de novo IBD in patients treated with Secukinumab was higher compared to the general population. Patients were young, obese, and prone to use the immunomodulator [81]. The third study was by Egeberg et al. in 2019, which followed a cohort of 235,038 Danish patients with psoriasis over a period of 20 years (each group of psoriasis patients had an associated group without psoriasis, as reference). The authors concluded that there was an association between IBD and the disease itself, as these patients were at increased risk of developing IBD. Patients receiving biologic therapy did not have a higher risk of developing IBD compared with the control group (biologic treatment was not separated/differentiated by drug class) [82].

Genome-wide association study (GWAS) showed that psoriasis and IBD have a common genetic background by sharing 13 common predisposing genes. The best known and studied loci are: 6p22 (CDKAL1), 16q (NOD2), 1p31 (IL-23 R), and 5q33 (IL12B). In addition to these, there are other common places, but less common: 20q13, 19p13, 6p21, and 5q31. Thus, it can be said that the two diseases are closely related by the overlapping of genetic factors and risk factors for the disease and by the cascade of proinflammatory cytokines [83,84].

Large-scale pharmacovigilance and epidemiological studies described the effect of SEC treatment on IBD. Table 3 shown some of the pharmacovigilance studies published in the past 4 years.

If Orell et al. and Egebergca et al. concluded that the incidence of de novo–IBD is low after SEC tratment, Mody et al. showed that de novo IBD incidence in SEC treated patients was higher in those who were younger and obese.

### 7.4. The Contribution of IL-17i to the Modification of the Intestinal Microbiome

The intestinal microbiome is a real universe and represents the totality of microorganisms that co-inhabit on the cell surface, which have important roles in the synthesis of vitamins, modulating the immune response, and defense against pathogens by it. The success of IL-17i therapy depends on the integrity of the intestinal microbiome, its alteration (intestinal dysbiosis) having a crucial role in the appearance of autoimmune diseases, chronic inflammatory arthritis (AS, PA), and also IBA [85]. Regarding IBD, it is reported in the literature that intestinal dysbiosis is due to an exacerbated or disordered immune response against common intestinal bacteria in people with genetic susceptibility.

In addition to their role in innate immunity, IL-17 inhibitors can also alter the intestinal microbiome, with expansions of *Candida albicans* and *Clostridium* species. The gastrointestinal tract is colonized by trillions of microorganisms, the intestinal immune response being closely correlated with them. The genus *Clostridium* plays an important role in intestinal homeostasis; by producing butyrate, it differentiates the regulatory T cells of the large intestine (T cell), which are essential for the prevention of autoimmune disease and self-tolerance [86,87,88]. Davidson et al. showed that in their study group, 12% of patients treated with Secukinumab required antifungal treatment compared to the group who received anti-IL12/23 or anti-TNFα treatment, thus proving that this treatment increases the risk of candidiasis, especially oropharyngeal and esophageal [42,89]. Under treatment with Ustekinumab, patients with Chron’s disease had higher bacterial diversity pre-treatment, while several taxa (e.g., *Faecalibacterium*) distinguished responders from non-responders [31]. Lepage et al. demonstrated the presence in the intestinal mucosa of patients with ulcerative colitis of a high percentage of *Actinobacteria* and *Proteobacteria* and a low percentage of *Bacteroides* [90].

Manasson et al. concluded in their study that the treatment of PA and AS with IL-17i, but also with other biological therapies, is associated with the disturbance of certain bacterial and fungal species (tax) and that the understanding of these changes would be the basis of precision medicine in AS, PA, and other rheumatic diseases [85]. Authors should discuss the results and how they can be interpreted from the perspective of previous studies and of the working hypotheses. The findings and their implications should be discussed in the broadest context possible. Future research directions may also be highlighted.

In summary, there are findings demonstrating that biologic therapies in PA and AS modulate immune cell response, but at the same time they are associated with dysbiosis. Moving forward, these IL-17i-induced microbial and immune perturbations should be explored, perhaps through a machine learning model, and incorporated into the clinic by predicting which individuals are susceptible to adverse outcomes from IL-17A and related therapies such as candidiasis and (sub)clinical gut inflammation. Ultimately, understanding the downstream effects of these perturbations could allow for the development of precision medicine approaches in PA, AS, and related rheumatic diseases.

## 8. Practical Recommendations

Research in recent years has led to a clearer understanding of the mechanism of IL-23-Th17 cell, IL-17, which has allowed for more targeted treatments and better control of psoriasis, PA, and AS. Thus, in psoriasis after treatment with IL-17i, epidermal hyperplasia improved, with 60% of patients having a full rate of skin cleansing [91]. Unlike psoriasis, in patients with IBD we have lesions of the intestinal epithelial layer. It is well known that there is a higher initial risk of developing IBD in patients with psoriasis, although the mechanism is not fully understood. Many patients with psoriasis may have subclinical IBD, which can be exposed with the use of IL-17 inhibitors. Physicians prescribing IL-17 treatment should be aware of new cases of IBD or its exacerbation so that patients can be properly examined and monitored for optimal results.

The onset of inflammatory intestinal disease may occur in patients with no gastrointestinal symptoms and no family history of inflammatory intestinal disease. Particular attention is recommended in the anamnesis for the identification of digestive symptoms in the personal history of patients and in the family history. Prior to initiating treatment with IL-17i, the existence of subclinical IBD by fecal calprotectin dosing should be ruled out. This is an inflammatory marker with a known role in inflammatory intestinal disease. Normal values (below 250 mcg/g) allow the start of treatment, and elevated values of calprotectin (above 250 mcg/g) require further investigation such as colonoscopy, abdominal, CT scan, MRE. If the presence of intestinal inflammation is confirmed, anti-IL-17 medication is contraindicated, with other therapeutic classes being preferred such as TNF inhibitors or corticosteroids [6,92].

## 9. Conclusions

IL-17 inhibitors are drugs used successfully in the treatment of dermatological and autoimmune diseases, but physicians should consider the possibility of the onset of IBD when recommending treatment with this category of drugs. The pathophysiological mechanism of these paradoxical reactions after IL-17i administration is not yet fully understood. Monitoring of patients during and after ceasing of IL-17 treatment is extremely important, as gastrointestinal symptoms are an alarm signal for the onset of inflammatory intestinal disease. The best management of these patients should involve a multidisciplinary team to identify possible adverse effects related to this class of medicines and to prevent complications.

## Figures and Tables

**Table 1:** Study methodology.

Identification of Studies via Databases and Registers for the Review	
Identification	Key words with “AND” operator “IL-17 inhibitors” AND ”inflammatory intestinal disease”
	Consulted databases Web of Science, PubMed, Scopus
	Criteria for inclusion Review or original article, relevant for the topic and thematic
	Criteria for exclusion Abstract paper, articles where full text was not available, not relevant to the topic or thematic
Records identified 170	
Articles after duplicate removal (duplicates = 28) 142	
Full-text analysis Appliance of inclusion and exclusioncriteria 106 records (eliminated 36 articles)	
Final bibliographical sources Verification and agreement on article 94 relevance and quality	

**Table 2:** Meta analysis studies and multiple case report about incidence and adverse effects of IL17i.

Study and Patients	Results	Observations	Reference
>56 million persons health records from Explorys * (IBM New York) Tracking period: 10 years (1999–2019)	At 6 months, the incidence of IBD: 0.16% among patients with Ps exposed to any IL-17i, 0.24% among those exposed to SEC alone, 0.11% among those unexposed. At first year: the incidence of IBD 0.27% for the patients who received IL-17i, 0.32% for those being treated with SEC only, 0.19% for those who did not receive treatment with IL-17i.	The incidence of IBD is low, and the risk appears similar to that in unexposed patients.	[68]
38 (RCTs **) including 16,690 patients Tracking period: 60 weeks	12 cases of new onset IBD were reported in five studies, whereas no cases were reported with placebo	De novo IBD was rare	[69]
21 RCT of 7355 patients with Ps, PsA, AS treated with SEC Tracking period: 5 years	Of the 5181 patients with Ps, 68 were diagnosed with IBD; of the 1380 with PsA, 8 cases of IBD were diagnosed, and of the 794 patients with AS, 13 developed IBD.	The IBD was present in 1.7%	[71]
66 studies of 14,390 patients exposed to induction and 19,380 patients exposed to induction and/or maintenance treatment Tracking period: 2010–2018	During induction, 11 cases of IBD were reported, 33 cases diagnosed during entire treatment period	The risk for onset of IBD in patients treated with IL-17i is not elevated	[72]
38 RCTs with a total 12,614 patients treated with IL-17i and 4076 treated with placebo. The RCTs included 8 studies of BRO (4588 patients), 8 of IXE (4485 patients), and 22 of SEC (7617 patients).	12 new cases of IBD (4 on IXE, 8 on SEC)	Incidence: 2.4 cases of new onset IBD/1000 patient-years. Statistically there was no difference in the risk of developing new onset IBD with IL-17i compared with placebo.	[14]
Collected clinical cases of de novo IBD reported in the studies on digestive paradoxical effects after treatment with IL-17i. Tracking period: up to 2020.	21 patients with IBD (19 after SEC and 2 after IXE).	The treatment with BRO, IXE, SEC, is associated with a low incidence of IBD in patients with rheumatological and dermatological autoimmune diseases in both clinical trials and clinical practice (2.4/1000 patient-years)	[6]
Published articles identifies 21 publications on the onset of IBD after treatment with IL-17Ai (SEC and IXE). Tracking period: 2018–2019	27 patients diagnosed with Ps, PsA, and AS after treatment with	IL-17i have proven efficacy for the treatment of Ps and PsA with a strong safety profile.	[4]

* Explorys = a multi-health system data analytics and research platform. ** RCT = randomized controlled trials.

**Table 3:** Pharmacovigilance studies.

Study and Patients	Results	Conclusion	Reference
>5 million patients with IBD treated with SEC and IXE from RADAR * and NMEDW. Tracking period: 2 years (2015–2017) for SEC IXE: 1 year and 7 months (2016–2017) for IXE	Cases with new IBD from reviewed database	AE reported after administration of SEC and IXE to the FAERS ** were insignificant. The PRR *** = 4.65	[81]
62 million electronic health records were analyzed from Explorys (IBM New York) ****. A number of 2870 patients were included	3.2% patients develop IBD after treatment with SEC; risk in population was 0.74%	The risk for de-novo IBD is higher compared to general population. The patients were obese and younger.	[82]
235,038 patients with Ps and non-Ps from the general population, all without a history of CD or UC at baseline Tracking period: 20 years	IBD appear in <1% of patients with Ps, but the biologic classes were not differentiated	Patients receiving biologic therapy did not have a higher risk of developing IBD compared with the control group (biologic treatement was not separated/differentiated by drug class)	[83]

* RADAR = Research on Adverse Drug events And Reports; ** FAERS = Food and Drug Administration Adverse Event Reporting System; *** PRR = the proportional reporting ratio; **** Explorys = a multi-health system data analytic and research platform.

## References

[1] Korn T., Bettelli E., Oukka M., Kuchroo V.K. (2009). IL-17 and Th17 Cells. Annu. Rev. Immunol..

[2] Onishi R.M., Gaffen S.L. (2010). Interleukin-17 and its target genes: Mechanisms of interleukin-17 function in dis-ease. Immunology.

[3] Draberova H., Janusova S., Knizkova D., Semberova T., Pribikova M., Ujevic A., Harant K., Knapkova S., Hrdinka M., Fanfani V. (2020). Systematic analysis of the IL-17 receptor signalosome reveals a robust regulatory feedback loop. EMBO Rep..

[4] Fieldhouse K.A., Ukaibe S., Crowley E.L., Khanna R., O’Toole A., Gooderham M.J. (2020). Inflammatory bowel disease in patients with psoriasis treated with interleukin-17 inhibitors. Drugs Context.

[5] Conrad C., Di Domizio J., Mylonas A., Belkhodja C., Demaria O., Navarini A.A., Lapointe A.K., French L.E., Vernez M., Gilliet M. (2018). TNF blockade induces a dysregulated type I interferon response without autoimmunity in paradoxical psoriasis. Nat. Commun..

[6] Fauny M., Moulin D., D’Amico F., Netter P., Petitpain N., Arnone D., Jouzeau J.Y., Loeuille D., Peyrin-Biroulet L. (2020). Paradoxical gastrointestinal effects of interleukin-17 blockers. Ann. Rheum. Dis..

[7] Brembilla N.C., Senra L., Boehncke W.-H. (2018). The IL-17 family of cytokines in psoriasis: IL-17A and beyond. Front. Immunol..

[8] Noviello D., Mager R., Roda G., Borroni R.G., Fiorino G., Vetrano S. (2021). The IL23-IL17 immune axis in the treatment of ulcerative colitis: Successes, defeats, and ongoing challenges. Front. Immunol..

[9] Yang X.O., Chang S.H., Park H., Nurieva R., Shah B., Acero L., Wang Y.-H., Schluns K.S., Broaddus R.R., Zhu Z. (2008). Regulation of inflammatory responses by IL-17F. J. Exp. Med..

[10] Li H., Chen J., Huang A., Stinson J., Heldens S., Foster J., Dowd P., Gurney A.L., Wood W.I. (2000). Cloning and characterization of IL-17B and IL-17C, two new members of the IL-17 cytokine family. Proc. Natl. Acad. Sci. USA.

[11] Im E., Jung J., Rhee S.H. (2012). Toll-like receptor 5 engagement induces interleukin-17C expression in intestinal epithelial cells. J. Interferon Cytokine Res..

[12] Reynolds J.M., Martinez G.J., Nallaparaju K.C., Chang S.H., Wang Y.H., Dong C. (2012). Cutting edge: Regulation of intestinal inflammation and barrier function by IL-17C. J. Immunol..

[13] Akimzhanov A.M., Yang X.O., Dong C. (2007). Chromatin remodeling of interleukin-17 (IL-17)-IL-17F cytokine gene locus during inflammatory helper T cell differentiation. J. Biol. Chem..

[14] Akiyama S., Sakuraba A. (2021). Distinct roles of interleukin-17 and T helper 17 cells among autoimmune diseases. J. Transl. Autoimmun..

[15] Berry G., Dossou S., Kashif C., Sharifinejad A., Azizi N., Hamedifar G., Sabzvari A., Zian A. (2022). The role of IL-17 and anti-IL-17 agents in the immunopathogenesis and management of autoimmune and inflammatory diseases. Int. Immunopharmacol..

[16] Gálvez J. (2014). Role of Th17 Cells in the Pathogenesis of Human IBD. ISRN Inflamm..

[17] Chang J.T. (2020). Pathophysiology of inflammatory bowel diseases. N. Engl. J. Med..

[18] Smillie C.S., Biton M., Ordovas-Montanes J., Sullivan K.M., Burgin G., Graham D.B., Herbst R.H., Rogel N., Slyper M., Waldman J. (2019). Intra- and inter-cellular rewiring of the human colon during ulcerative colitis. Cell.

[19] Garduño R.C., Däbritz J. (2021). New Insights on CD8+ T cells in inflammatory bowel disease and therapeutic Approaches. Front. Immunol..

[20] Amatya N., Garg A.V., Gaffen S.L. (2017). IL-17 Signaling: The Yin and the Yang. Trends Immunol..

[21] Maloy K.J., Kullberg M.C. (2008). IL-23 and Th17 cytokines in intestinal homeostasis. Mucosal Immunol..

[22] Ogura H., Murakami M., Okuyama Y., Tsuruoka M., Kitabayashi C., Kanamoto M., Nishihara M., Iwakura Y., Hirano T. (2008). Interleukin-17 promotes autoimmunity by triggering a positive-feedback loop via interleukin-6 induction. Immunity.

[23] Shalom-Barak T., Quach J., Lotz M. (1998). Interleukin-17-induced gene expression in articular chondrocytes is associated with activation of mitogen-activated protein kinases and NF-kappaB. J. Biol. Chem..

[24] Rouvier E., Luciani M.F., Mattéi M.G., Denizot F., Golstein P. (1993). CTLA-8, cloned from an activated T cell, bearing AU-rich messenger RNA instability sequences, and homologous to a Herpesvirus Saimiri Gene. J. Immunol..

[25] Gaffen S.L. (2008). An Overview of IL-17 function and signaling. Cytokine.

[26] Yao Z., Painter S.L., Fanslow W.C., Ulrich D., Macduff B.M., Spriggs M.K., Armitage R.J. (1995). Cutting edge: Human IL-17: A novel cytokine derived from T cells. J. Immunol..

[27] Liu C., Qian W., Qian Y., Giltiay N.V., Lu Y., Swaidani S., Misra S., Deng L., Chen Z.J., Li X. (2009). Act1, a U-Box E3 ubiquitin ligase for IL-17 signaling. Sci. Signal..

[28] Schwandner R., Yamaguchi K., Cao Z. (2000). Requirement of tumor necrosis factor receptor-associated factor (TRAF) 6 in interleukin 17 signal transduction. J. Exp. Med..

[29] Song X., Qian Y. (2013). The activation and regulation of IL-17 receptor mediated signaling. Cytokine.

[30] Sun D., Novotny M., Bulek K., Liu C., Li X., Hamilton T. (2011). Treatment with IL-17 prolongs the half-life of chemokine CXCL1 mRNA via the adaptor TRAF5 and the splicing-regulatory factor SF2 (ASF). Nat. Immunol..

[31] Herjan T., Yao P., Qian W., Li X., Liu C., Bulek K., Sun D., Yang W.P., Zhu J., He A. (2013). HuR is required for IL-17-induced Act1-mediated CXCL1 and CXCL5 mRNA stabilization. J. Immunol. Res..

[32] Zenobia C., Hajishengallis G. (2000). Basic biology and role of interleukin-17 in immunity and inflammation. Periodontology.

[33] Boyle W.J., Simonet W.S., Lacey D.L. (2003). Osteoclast differentiation and activation. Nature.

[34] Kwan W.H., van der Touw W., Paz-Artal E., Li M.O., Heeger P.S. (2013). Signaling through C5a receptor and C3a receptor diminishes function of murine natural regulatory T cells. J. Exp. Med..

[35] Miossec P., Kolls J.K. (2012). Targeting IL-17 and TH17 cells in chronic inflammation. Nat. Rev. Drug Discov..

[36] Sarkar S., Justa S., Brucks M., Endres J., Fox D.A., Zhou X., Alnaimat F., Whitaker B., Wheeler J.C., Jones B.H. (2014). Interleukin (IL)-17A, F and AF in inflammation: A study in collagen-induced arthritis and rheumatoid arthritis: IL-17 subtypes in inflammatory arthritis. Clin. Exp. Immunol..

[37] Patel D.D., Lee D.M., Kolbinger F., Antoni C. (2013). Effect of IL-17A blockade with Secukinumab in autoimmune diseases. Ann. Rheum. Dis..

[38] Gaston J.S.H., Jadon D.R. (2017). Th17 cell responses in spondyloarthritis. Best Pract. Res. Clin. Rheumatol..

[39] Jandus C., Bioley G., Rivals J.-P., Dudler J., Speiser D., Romero P. (2008). Increased numbers of circulating polyfunctional Th17 memory cells in patients with seronegative spondylarthritides. Arthritis Rheum..

[40] Blanco F.J., Möricke R., Dokoupilova E., Codding C., Neal J., Andersson M., Rohrer S., Richards H. (2017). Secukinumab in active rheumatoid arthritis: A phase III randomized, double-blind, active comparator- and placebo-controlled study. Arthritis Rheumatol..

[41] Appel H., Maier R., Wu P., Scheer R., Hempfing A., Kayser R., Thiel A., Radbruch A., Loddenkemper C., Sieper J. (2011). Analysis of IL-17^+^ cells in facet joints of patients with spondyloarthritis suggests that the innate immune pathway might be of greater relevance than the Th17-mediated adaptive immune response. Arthritis Res. Ther..

[42] Jones A., Ciurtin C., Ismajli M., Leandro M., Sengupta R., Machado P.M. (2018). Biologics for treating axial spondyloarthritis. Expert Opin. Biol. Ther..

[43] Lucaciu L.A., Ilieș M., Vesa Ștefan C., Seicean R., Din S., Iuga C.A., Seicean A. (2021). Serum interleukin (IL)-23 and IL-17 profile in inflammatory bowel disease (IBD) patients could differentiate between severe and non-severe disease. J. Pers. Med..

[44] Pallag A., Rosca E., Tit D.M., Mutiu G., Bungau S.G., Pop O.L. (2015). Monitoring the effects of treatment in colon cancer cells using immunohistochemical and histoenzymatic techniques. Rom. J. Morphol. Embriol..

[45] Mirsattari D., Seyyedmajidi M., Zojaji H., Haghazali M., Orimi P.G., Shoushtarizadeh T., Almasi S. (2012). The relation between the level of interleukin-23 with duration and severity of ulcerative colitis. Gastroenterol. Hepatol. Bed Bench.

[46] Fujino S., Andoh A., Bamba S., Ogawa A., Hata K., Araki Y., Bamba T., Fujiyama Y. (2003). Increased expression of interleukin 17 in inflammatory bowel disease. Gut.

[47] Jiang W., Su J., Zhang X., Cheng X., Zhou J., Shi R., Zhang H. (2014). Elevated levels of Th17 cells and Th17-related cytokines are associated with disease activity in patients with inflammatory bowel disease. Inflamm. Res..

[48] Gheita T.A., El Gazzar I.I., El-Fishawy H.S., Aboul-Ezz M.A., Kenawy S.A. (2012). Involvement of IL-23 in enteropathic arthritis patients with inflammatory bowel disease: Preliminary results. Gastroenterol. Hepatol..

[49] Fuss I.J., Becker C., Yang Z., Groden C., Hornung R.L., Heller F., Neurath M.F., Strober W., Mannon P.J. (2006). Both IL-12p70 and IL-23 are synthesized during active Crohn’s disease and are down-regulated by treatment with anti-IL-12 P40 monoclonal antibody. Inflamm. Bowel Dis..

[50] McGovern D., Powrie F. (2007). The IL23 axis plays a key role in the pathogenesis of IBD. Gut.

[51] Duerr R.H., Taylor K.D., Brant S.R., Rioux J.D., Silverberg M.S., Daly M.J. (2006). A genome-wide association study identifies IL-23R as an inflammatory bowel disease gene. Science.

[52] Einarsdottir E., Koskinen L.L.E., Dukes E., Kainu K., Suomela S., Lappalainen M., Ziberna F., Korponay-Szabo I.R., Kurppa K., Kaukinen K. (2009). IL23R in the Swedish, Finnish, Hungarian and Italian populations: Association with IBD and psoriasis, and linkage to celiac disease. BMC Med. Genet..

[53] Ciccia F., Guggino G., Rizzo A., Saieva L., Peralta S., Giardina A., Cannizzaro A., Sireci G., De Leo G., Alessandro R. (2015). Type 3 innate lymphoid cells producing IL-17 and IL-22 are expanded in the gut, in the peripheral blood, synovial fluid and bone marrow of patients with ankylosing spondylitis. Ann. Rheum. Dis..

[54] Zeng B., Shi S., Ashworth G., Dong C., Liu J., Xing F. (2019). ILC3 Function as a double-edged sword in inflammatory bowel diseases. Cell Death Dis..

[55] Allocca M., Furfaro F., Fiorino G., Gilardi D., D’Alessio S., Danese S. (2018). Can IL-23 be a good target for ulcerative colitis?. Best Pract. Res. Clin. Gastroenterol..

[56] Okayasu I., Hatakeyama S., Yamada M., Ohkusa T., Inagaki Y., Nakaya R. (1990). A novel method in the induction of reliable experimental acute and chronic ulcerative colitis in mice. Gastroenterology.

[57] Ogawa A., Andoh A., Araki Y., Bamba T., Fujiyama Y. (2004). Neutralization of interleukin-17 aggravates dextran sulfate sodium-induced colitis in mice. Clin. Immunol..

[58] GBD 2017 Inflammatory Bowel Disease Collaborators (2020). The global, regional, and national burden of inflammatory bowel disease in 195 countries and territories, 1990–2017: A systematic analysis for the global burden of disease study 2017. Lancet Gastroenterol. Hepatol..

[59] Khor B., Gardet A., Xavier R.J. (2011). Genetics and pathogenesis of inflammatory bowel disease. Nature.

[60] Däbritz J., Gerner P., Enninger A., Claßen M., Radke M. (2017). Inflammatory bowel disease in childhood and adolescence. Dtsch. Arztebl. Int..

[61] Negrut N., Khan S.A., Bungau S., Zaha D.C., Anca C.A.R., Bratu O., Diaconu C.C., Ionita-Radu F. (2020). Diagnostic challenges in gastrointestinal infections. Rom. J. Mil. Med..

[62] Petitpain N., D’Amico F., Yelehe-Okouma M., Jouzeau J.-Y., Netter P., Peyrin-Biroulet L., Gillet P. (2021). IL-17 inhibitors and inflammatory bowel diseases: A postmarketing study in Vigibase. Clin. Pharmacol. Ther..

[63] Cosentyx Prescribing Information; Novartis Pharmaceutical Corporation: Basel, Switzerland. https://www.pharma.us.novartis.com/sites/www.pharma.us.novartis.com/files/cosentyx.pdf.

[64] Hohenberger M., Cardwell L.A., Oussedik E., Feldman S.R. (2018). Interleukin-17 inhibition: Role in psoriasis and inflammatory bowel disease. J. Dermatolog. Treat..

[65] Langley R.G., Elewski B.E., Lebwohl M., Reich K., Griffiths C.E., Papp K., Puig L., Nakagawa H., Spelman L., Sigurgeirsson B. (2014). ERASURE Study Group; FIXTURE Study Group. Secukinumab in plaque psoriasis-Results of two phase 3 Trials. N. Engl. J. Med..

[66] Hueber W., Sands B.E., Lewitzky S., Vandemeulebroecke M., Reinisch W., Higgins P.D.R., Wehkamp J., Feagan B.G., Yao M.D., Karczewski M. (2012). Secukinumab, a human anti-IL-17A monoclonal antibody, for moderate to severe Crohn’s disease: Unexpected results of a randomised, double-blind placebo-controlled trial. Gut.

[67] Gómez A.O., Velázquez L.M., Sanchez L.B., Sánchez I.P., Rabasco E.P., Monsalve G., Ferrández A.G., Sepulcre J. (2021). Inflammatory bowel disease new-onset during Secukinumab therapy: Real-world data from a tertiary center. Rev. Esp. Enferm. Dig..

[68] Wright S., Aloo A., Strunk A., Garg A. (2020). Real-world risk of new-onset inflammatory bowel disease among patients with psoriasis exposed to interleukin 17 inhibitors. J. Am. Acad. Dermatol..

[69] Yamada A., Wang J., Komaki Y., Komaki F., Micic D., Sakuraba A. (2019). Systematic review with meta-analysis: Risk of new onset IBD with the use of anti-interleukin-17 agents. Aliment. Pharmacol. Ther..

[70] Wang J., Bhatia A., Krugliak Cleveland N., Gupta N., Dalal S., Rubin D.T., Sakuraba A. (2018). Rapid onset of inflammatory bowel disease after receiving Secukinumab infusion. ACG Case Rep. J..

[71] Schreiber S., Colombel J.F., Feagan B.G., Reich K., Deodhar A.A., McInnes I.B., Porter B., Das Gupta A., Pricop L., Fox T. (2019). Incidence rates of inflammatory bowel disease in patients with psoriasis, psoriatic arthritis and ankylosing spondylitis treated with Secukinumab: A retrospective analysis of pooled data from 21 clinical trials. Ann. Rheum. Dis..

[72] Burisch J., Eigner W., Schreiber S., Aletaha D., Weninger W., Trauner M., Reinisch W., Narula N. (2020). Risk for development of inflammatory bowel disease under inhibition of interleukin 17: A systematic review and meta-analysis. PLoS ONE.

[73] Griffiths C., Hardin D.S., Abreu M.T., Sartor B., Xu R., Solotkin W., Bachelez K., Colombel H. (2017). Incidence of inflammatory bowel disease among Ixekizumab-treated patients with moderate-to-severe plaque psoriasis and psoriatic arthritis: Data from 8 Clinical Trials. J. Am. Acad. Dermatol..

[74] Smith M.K., Pai J., Panaccione R., Ferraz J.G., Jijon J.G. (2019). Crohn’s-like disease in a patient exposed to anti-Interleukin-17 blockade (Ixekizumab) for the treatment of chronic plaque psoriasis: A case report. BMC Gastroenterol..

[75] Van Der Heijde D., Wei C.-C., Dougados J., Mease M., Deodhar P., Maksymowych A., Van Den Bosch W.P., Sieper F., Tomita J., Landewé T. (2018). Carlier on Behalf of the COAST-V Study Group* Ixekizumab, an Interleukin-17A Antagonist in the Treatment of Ankylosing Spondylitis or Radiographic Axial Spondyloarthritis in Patients Previously Untreated with Biological Disease-Modifying Anti-Rheumatic Drugs (COAST-V): 16 Week Results of a Phase 3 Randomised, Double-Blind, Active-Controlled and Placebo-Controlled Trial. Lancet.

[76] Strober B., Leonardi C., Papp K.A., Mrowietz U., Ohtsuki M., Bissonnette R., Ferris L.K., Paul C., Lebwohl M., Braun D.K. (2017). Short- and long-term safety outcomes with Ixekizumab from 7 clinical trials in psoriasis: Etanercept comparisons and integrated Data. J. Am. Acad. Dermatol..

[77] Papp K.A., Leonardi C., Menter A., Ortonne J.P., Krueger J.G., Kricorian G., Aras G., Li J., Russell C.B., Thompson E.H.Z. (2012). Brodalumab, an anti-interleukin-17-receptor antibody for psoriasis. N. Engl. J. Med..

[78] Targan S.R., Feagan B., Vermeire S., Panaccione R., Melmed G.Y., Landers C., Li D., Russell C., Newmark R., Zhang N. (2016). A Randomized, double-blind, placebo-controlled phase 2 study of Brodalumab in patients with moderate-to-severe Crohn’s disease. Am. J. Gastroenterol..

[79] Ritchlin C.T., Kavanaugh A., Merola J.F., Schett G., Scher J.U., Warren R.B., Assudani D., Kumke T., Ink B., McInnes I.B. (2018). Dual neutralization of IL-17A and IL-17F with bimekizumab in patients with active PsA: Results from a 48-week phase 2b, randomized, double-blind, placebo-controlled, dose-ranging study. Arthritis Rheumatol..

[80] Orrell K.A., Murphrey M., Kelm R.C., Lee H.H., Pease D.R., Laumann A.E., West D.P., Nardone B. (2018). Inflammatory bowel disease events after exposure to interleukin 17 inhibitors Secukinumab and Ixekizumab: Postmarketing analysis from the RADAR (“Research on Adverse Drug Events and Reports”) Program. J. Am. Acad. Dermatol..

[81] Mohy-ud-din N., Carleton N., El-Hachem S., Baki H.A., Syed A., Dulai P., Kochhar G. (2019). 731 De Novo Inflammatory Bowel Disease after Secukinumab Use: A Population Based Analysis: 731. Am. J. Gastroenterol..

[82] Jancin B. Here Comes Bimekizumab, the Newest IL-17 Inhibitor. https://www.mdedge.com/edermatologynews/article/158562/psoriatic-arthritis/here-comes-bimekizumab-newest-Il-17-inhibitor.

[83] Egeberg A., Thyssen J.P., Burisch J., Colombel J.-F. (2019). Incidence and Risk of Inflammatory Bowel Disease in Patients with Psoriasis-A Nationwide 20-Year Cohort Study. J. Investig. Dermatol..

[84] Maronese C.A., Zelin E., Moltrasio C., Genovese G., Marzano A.V. (2021). Genetic screening in new onset inflammatory bowel disease during anti-interleukin 17 therapy: Unmet needs and call for action. Expert Opin. Biol. Ther..

[85] Manasson L., Wallach D.S., Guggino G., Stapylton M., Badri M.B., Solomon G., Reddy S.M., Aksenov C.R., Jones A.A., Girija D.R. (2020). Interleukin-17 Inhibition in Spondyloarthritis Is Associated with Subclinical Gut Microbiome Perturbations and a Distinctive Interleukin-25-Driven Intestinal Inflammation. Arthritis Rheumatol..

[86] Vlachos C., Gaitanis G., Katsanos K.H., Christodoulou D.K., Tsianos E., Bassukas I.D. (2016). Psoriasis and inflammatory bowel disease: Links and risks. Psoriasis.

[87] Lopetuso L.R., Scaldaferri F., Petito V., Gasbarrini A. (2013). Commensal Clostridia: Leading players in the maintenance of gut homeostasis. Gut Pathog..

[88] Furusawa Y., Obata Y., Fukuda S., Endo T.A., Nakato G., Takahashi D., Nakanishi Y., Uetake C., Kato K., Kato T. (2013). Commensal microbe-derived butyrate induces the differentiation of colonic regulatory T cells. Nature.

[89] Davidson L., van den Reek J.M.P.A., Bruno M., van Hunsel F., Herings R.M.C., Matzaraki V., Boahen C.K., Kumar V., Groenewoud H.M.M., van de Veerdonk F.L. (2022). Risk of candidiasis associated with interleukin-17 inhibitors: A real-world observational study of multiple in-dependent sources. Lancet Reg. Health Eur..

[90] Blauvelt A., Chiricozzi A. (2018). The immunologic role of IL-17 in psoriasis and psoriatic arthritis pathogenesis. Clin. Rev. Allergy Immunol..

[91] D’Amico F., Bonovas S., Danese S., Peyrin-Biroulet L. (2020). Review Article: Faecal Calprotectin and Histologic Remission in Ulcerative Colitis. Aliment. Pharmacol. Ther..

[92] Lepage P., Häsler R., Spehlmann M.E., Rehman A., Zvirbliene A., Begun A., Ott S., Kupcinskas L., Doré J., Raedler A. (2011). Twin study indicates loss of interaction between microbiota and mucosa of patients with ulcerative colitis. Gastroenterology.

